# Editorial: The Interplay Between Sleep and Emotion: What Role Do Cognitive Processes Play?

**DOI:** 10.3389/fpsyg.2020.612498

**Published:** 2020-11-30

**Authors:** Nicola Cellini, Caterina Lombardo

**Affiliations:** ^1^Department of General Psychology, University of Padova, Padua, Italy; ^2^Department of Biomedical Sciences, University of Padova, Padua, Italy; ^3^Padova Neuroscience Center, University of Padova, Padua, Italy; ^4^Human Inspired Technology Center, University of Padova, Padua, Italy; ^5^Department of Psychology, Sapienza University of Rome, Rome, Italy

**Keywords:** sleep, insomnia, emotion, memory, cognition

Sleep is an intrinsic condition of life. Although “why do we sleep” is still an elusive question, in the last decade several studies have shown that sleep plays a key role in memory and emotional processing. Yet, the specific mechanisms underlying sleep-related memory and emotional processes remain partially unknown, and studies often report contradictory results (Cordi and Rasch, 2020). From a psychopathology point of view, literature is showing how people suffering from sleep problems, such as in insomnia, are often characterized by cognitive dysfunctions and emotion dysregulation (Jansson-Fröjmark et al., 2016; Cellini, 2017; Ballesio et al., 2018). Neuroimaging studies are also supporting this view. As recently summarized by Schiel et al. (2020), insomnia is related to altered amygdala reactivity, morphometry, and adaptation. Moreover, insomnia is associated with aberrant connectivity in the default mode network, alterations in the salience network associated with hyperarousal, maladaptive emotion regulation, and disturbed integration of emotional states. Considering that the ability to adaptively regulate emotions is crucial for healthy functioning, the dysregulation of negative affect circuitry associated or due to insomnia may mediate the impact of insomnia on psychopathology. Indeed, sleep difficulties are often associated with other mental disorders and may be considered a transdiagnostic risk factor (Fairholme et al., 2013), although the role of the mediating factors still needs to be clarified.

The studies collected in the present Research Topic contribute to shed light on the relationship between sleep, cognitive, and emotional processing in healthy and clinical populations, discussing the most advanced development in the field from different perspectives (e.g., neurophysiological, behavioral, clinical). There are nine manuscripts in this special issue, including seven original research studies and two meta-analyses targeting two different aspects of the relationship between sleep and emotions.

Four papers focused their attention on insomnia. Feige et al. addressed the role of physiological arousal showing how evening relaxation positively influences sleep architecture, in particular in patients with insomnia disorders, suggesting how relaxation may be a useful therapeutic target in conjunction with other treatments for insomnia. In their opinion paper, Akram et al. focused on the cognitive aspects of insomnia, discussing, in particular, the relationship between attentional bias for sleep-related threat information and insomnia. The authors propose several candidate factors, including mood state and worry, that may influence the sleep-related attentional biases in people suffering from insomnia. The authors also discuss the potential beneficial role of attentional bias modification training for treating insomnia symptomatology. The study of de Almondes et al. investigates the relationship between facial emotion recognition and executive functioning among individuals with insomnia. Results indicate that patients with insomnia disorders show both a lower facial recognition of fear and sadness and impaired executive functions (i.e., inhibitory control, planning capacity, problem-solving, and cognitive flexibility) compared to healthy controls. Impairment in executive functions in individuals with insomnia is also supported by the meta-analysis conducted by Ballesio et al. The authors conducted a systematic review of the literature on three components of executive functions (i.e., inhibitory control, working memory, and cognitive flexibility). Results of the 28 studies included in the meta-analysis indicate small to moderate deficits in executive functions in individuals with insomnia.

Three other papers focused their attention on different aspects of emotional memory in relation to sleep. Jones et al. examined the impact of emotional memory processing during a daytime nap on people's moods. They showed that positive affect decreases after viewing unpleasant pictures, but it recovers after a daytime nap as a function of the sigma activity level. Moreover, the recovery seems to be moderated by the level of emotional memory consolidation, suggesting an interesting relationship between the processing of emotional memories during sleep and post-sleep affect. The study by Cellini et al. investigated the fate of emotional memories over 1 week. Their results indicate that emotional memories are resistant to forgetting, particularly when sleep is disrupted. Moreover, their results indicate that emotional memory consolidation over time seems not to be affected by non-clinical depression symptomatology. The relationship between sleep and emotional memory consolidation is also the focus of the meta-analysis conducted by Lipinska et al.. Analyzing data from 31 articles, the authors showed that sleep preferentially consolidates emotional over neutral memories, but only under specific conditions. For example, studies using free-recall show a more robust effect than studies employing recognition memory tasks.

The remaining two studies targeted different aspects of sleep and emotion/cognition. Ackermann et al. tested the effect of psychosocial stress on subsequent sleep characteristics and cognitive functioning. Using a daytime nap paradigm, they showed that psychosocial stress increases sleep latency and reduce slow-wave activity (i.e., a marker of deep sleep), but the latter effect was short-lasting. Moreover, the stressful situation before a nap did not impair cognitive functioning. Lastly, Simor et al. investigated the time curse of sequence and statistical learning, showing that the knowledge acquired during training is preserved after an off-line period regardless of the presence of sleep, although sleep oscillations seem to modulate memory consolidation at the individual level.

## Conclusions

The papers included in this Research Topic contributed to shed light on the complex relationship, characterized by reciprocal influences, between sleep and emotional and cognitive processing. They confirm that the alterations of sleep (e.g., due to the chronic effect of insomnia) have a negative impact on cognitive and emotional functioning. However, they also support the negative impact of cognitive and emotional processes on sleep thus suggesting the reciprocal influences of those two domains pertaining to a cycle that only conventionally is divided into wake (cognitive and emotional functioning) and sleep (quality or duration). Nevertheless, more research is needed to further advance our understandings of the relationship between sleep and cognitive and emotional processing, in particular in light of the fundamental role of sleep on physical and mental health. Further studies are also required to better understand the factors that may mediate or moderate the impact of chronic alteration of sleep on psychological functioning and mental health.

## Author Contributions

NC and CL have made a substantial, direct and intellectual contribution to the work and approved it for publication.

## Conflict of Interest

The authors declare that the research was conducted in the absence of any commercial or financial relationships that could be construed as a potential conflict of interest.
